# Extracellular vesicles through the blood–brain barrier: a review

**DOI:** 10.1186/s12987-022-00359-3

**Published:** 2022-07-25

**Authors:** Héctor M. Ramos-Zaldívar, Iva Polakovicova, Edison Salas-Huenuleo, Alejandro H. Corvalán, Marcelo J. Kogan, Claudia P. Yefi, Marcelo E. Andia

**Affiliations:** 1 Doctoral Program in Medical Sciences, Faculty of Medicine, Pontificia Universidad Catolica de Chile, Santiago de Chile, Chile; 2 Advanced Center for Chronic Diseases, Santiago, Chile; 3 Department of Hematology and Oncology, Faculty of Medicine, Pontificia Universidad Católica de Chile, Santiago, Chile; 4 Advanced Integrated Technologies SpA, (AINTECH), Santiago, Chile; 5 Departamento de Química Farmacológica Y Toxicológica, Facultad de Ciencias Químicas Y Farmacéuticas, Laboratorio de Nanobiotecnología, Universidad de Chile, Carlos Lorca 964, Independencia, Chile; 6 Escuela de Medicina Veterinaria, Facultad de Agronomía E Ingeniería Forestal, Facultad de Ciencias Biológicas Y Facultad de Medicina, Pontificia Universidad Católica de Chile, Santiago, Chile; 7 Biomedical Imaging Center, School of Medicine, Pontificia Universidad Católica de Chile, Santiago, Chile; 8 Millennium Institute for Intelligent Healthcare Engineering, Santiago, Chile

**Keywords:** Extracellular vesicles, Blood–Brain barrier, Transcytosis, Exosomes

## Abstract

Extracellular vesicles (EVs) are particles naturally released from cells that are delimited by a lipid bilayer and are unable to replicate. How the EVs cross the Blood–Brain barrier (BBB) in a bidirectional manner between the bloodstream and brain parenchyma remains poorly understood. Most in vitro models that have evaluated this event have relied on monolayer transwell or microfluidic organ-on-a-chip techniques that do not account for the combined effect of all cellular layers that constitute the BBB at different sites of the Central Nervous System. There has not been direct transcytosis visualization through the BBB in mammals in vivo, and evidence comes from in vivo experiments in zebrafish. Literature is scarce on this topic, and techniques describing the mechanisms of EVs motion through the BBB are inconsistent. This review will focus on in vitro and in vivo methodologies used to evaluate EVs transcytosis, how EVs overcome this fundamental structure, and discuss potential methodological approaches for future analyses to clarify these issues. Understanding how EVs cross the BBB will be essential for their future use as vehicles in pharmacology and therapeutics.

## Background

Extracellular vesicles (EVs) are particles delineated by a lipid bilayer that are naturally released from cells but cannot replicate [1]. Although microvesicles, exosomes, and apoptotic bodies are usually regarded as the three main subtypes of EVs, classification varies upon biogenesis, release pathway, size, content, and function [2]. Other nomenclature can be found in the literature based on whether they have an endosomal origin (exosomes) or are derived from the plasma membrane (ectosomes, microparticles, microvesicles) [1, 3]. Furthermore, the Minimal Information for Studies of Extracellular Vesicles 2018 (MISEV2018), has proposed the use of operational terms based on the physical characteristics of EVs, such as size (“small EVs” < 200 nm and “medium/large EVs” > 200 nm); their biochemical composition (CD63 + /CD81 +—EVs, Annexin A5-stained EVs, etc.); or descriptions of conditions or cell of origin (podocyte EVs, hypoxic EVs, large oncosomes, apoptotic bodies) [1]. EVs show roles in diverse processes such as intercellular communication, transportation of biological contents, homeostasis, and cellular response to environmental changes on the parent and recipient cells [4–7].

The relevance of EVs research in the past two decades has increased exponentially, as demonstrated by a 733-fold rise in publication output since the year 2000 [8]. This is mainly due to their potential diagnostic and therapeutic applications in fields such as cancer, neurodegenerative, and immunological diseases [9–11]. For example, a phase I study by Escudier et al*.* that used autologous Dendritic Cell-Derived EVs (DEX) loaded with Melanoma-associated antigen 3 (MAGE-3) as immunotherapy in metastatic melanoma patients showed no major toxicity, a partial response, and tumor regressions at skin and lymph node sites [12]. Other phase I and phase II studies have confirmed these antitumor effects of DEX and MAGE-3 on advanced non-small cell lung cancer patients[13, 14]; ascites-derived EVs combined with granulocyte–macrophage colony-stimulating factor (GM-CSF) on colorectal cancer[15]; tumor cell-derived EVs packed with methotrexate in lung cancer [16]; and even plant-derived EVs loaded with curcumin in colon cancer (NCT01294072) [17]. In Alzheimer Disease (AD), possible biomarkers with diagnostic relevance have been identified, such as elevated lysosome-associated membrane protein 1 (LAMP1) and cathepsin D levels in plasma EVs [18], and low levels of EV-associated miRNA-193b in cerebrospinal fluid [19, 20]. In immunology, EVs derived from mesenchymal stem cells and dendritic cells have shown positive results in reduction of inflammation and promotion of tissue regeneration in preclinical models of osteoarthritis, autoimmune uveitis, and Irritable Bowel Syndrome [21–24]. Most recently, EVs research has also played a role in developing vaccines against COVID-19, by expressing the SARS-CoV-2 Spike protein on their surface or by delivering mRNAs of viral proteins [25].

However, as highlighted by Margolis et al., many concepts remain obscure with untested hypotheses and speculations awaiting experimental proofs [26]; including implications of size diversity, biogenesis pathways, and surface characteristics on biological effects, targeting, and cell physiology [26]. One of the most outstanding unsolved issues to date regarding EVs lies in their ability to cross biological barriers and punctually the blood–brain barrier (BBB) in a bidirectional manner to influence either neurons or peripheral tissues through the bloodstream. A more detailed comprehension on this phenomenon becomes particularly important when considering the potential for the use of EVs as vehicles in pharmacology and therapeutics. In the last 10 years advances have been made in the evaluation of the therapeutic effects of EVs in pre-clinical models of brain diseases such as AD, stroke, traumatic brain injury, and intracerebral hemorrhage [27]. Unfortunately, there is still inconsistency in the scarce literature available that evaluates the mechanisms for EVs crossing of the BBB. This review will discuss in vitro and in vivo methodologies that have been implemented to examine EVs transcytosis through the BBB, as well as possible future perspectives that could contribute to its analysis.

## In vitro models to study EVs crossing the blood–brain barrier

### BBB models

Different versions of the Boyden Chamber assay have been applied to assess migration of EVs through a BBB model. Nevertheless, simulation of the BBB layer has differed substantially among experiments, most evaluating monolayer models derived from different species. Permeability assays to test the tight junctions integrity at the BBB have included transendothelial electrical resistance (TEER) [28, 29], use of 70 kDa Fluorescein isothiocyanate (FITC)-dextran [30–32], 10 kDa dextran-Alexa 647 [31], and sodium fluorescein [29].

A comparison of transwell models used to evaluate EVs crossing through the BBB is shown in Table 1. Chen et al. used a human brain microvascular endothelial cell (BMEC) monolayer grown for 48 h on type I collagen-coated 6.5 mm transwell culture inserts with a pore size of 0.45 µm [30]. BMECs were obtained from an American Type Culture Collection (ATCC) cell line, expanded in endothelial cell growth medium (Lonza) and supplemented with SingleQuot Kit Supplements and growth factors (Lonza) [30]. Morad et al. also used a monolayer consisting of primary human BMECs (Cell Systems Co.) cultured for 48 h but on 0.4 μm pore polycarbonate membrane inserts coated with 50 μg/mL human plasma fibronectin [31]. Then BMECs were fed endothelial growth media supplemented with 8-(4-chlorophenylthio)- adenosine 3′,5′-cyclic monophosphate, and 4-(3-butoxy-4-methoxybenzyl)-2-imidazolidinone [31]. Matsumoto et al. employed cells from a different species, constructing a monolayer of primary CD1-mice BMECs (4 × 10^4^ cells/well) seeded onto fibronectin and collagen type IV pre-coated transwell inserts of 0.33 cm^2^, 0.4-μm pore size. Medium with hydrocortisone (500 nM) was added to reinforce tight junctions [28].

**Table 1:** Transwell models used to evaluate crossing of EVs through the BBB

Author	Type of cells	Number of layers at crossing	Time grown	Coated with	Pore size	Additional internvention
Chen et al	Human BMECs	Monolayer	48 h	Collagen type 1	0.45 µm	N.A
Morad et al	Human BMECs	Monolayer	48 h	Fibronectin	0.40 µm	8-CPT-cAMP and RO 20–1724
Matsumoto et al	CD1 mice BMECs	Monolayer	N.A	Fibronectin and collagen type IV	0.40 µm	Hydrocortisone
Tominaga et al	Monkey BMECs and Wistar rat pericytes	Bilayer	N.A	N.A	3.00 µm	Hydrocortisone

*BMECs* brain microvascular endothelial cells, *N.A.* information not available.

BMEC monolayer experiments have resulted in essential descriptions on potential interactions of EVs with the endothelial component of the BBB. Still, the mechanisms proposed for these vesicles to reach the brain parenchyma could be insufficient to establish the whole process clearly. The BBB is also supported by mural cells comprising of vascular smooth muscle cells and pericytes, as well as glial astrocytic cells that contribute to the regulation of components that ultimately reach neurons [33]. These have rarely been considered in BBB transwell experiments evaluating EVs. To the best of our knowledge, Tominaga et al. is the only group that has considered using a BBB kit made of primary cultures of monkey *Macaca irus* brain capillary endothelial cells with an added Wistar rat pericyte layer before reaching Wistar rat astrocytes at the base of the well (according to MBT-24H PharmaCo-Cell supplier description linked by the authors) [29]. Still, astrocyte crossing was not addressed. The MBT-24H BBB kit consists of a larger 3.0 µm pore size insert and describes the use of hydrocortisone supplementation [34].

Another approach was implemented by Morad et al. with a microfluidic organ-on-a-chip model of the BBB. This consisted of a 2-channel microfluidic culture containing a vascular channel lined by induced pluripotent stem cell-derived human microvascular endothelial cells, separated by a porous extracellular matrix-coated membrane from an abluminal channel containing primary human astrocytes and pericytes [31, 35]. Fluorescence microscopy analyses showed the presence of EVs that were taken up by astrocytes in the abluminal chamber, demonstrating that EVs can interact with endothelial cells under flow conditions and continuously cross the endothelial monolayer [31]. However, this still focuses only on the endothelial cell crossing, measuring the amount of signal reaching astrocytes and pericytes but without elucidating a subsequent passing through a pericyte-astrocyte bilayer.

Monolayer techniques contribute valuable information on the interaction of EVs with different components of the BBB, giving the primary close-up about the interaction with BBB. However, they do not necessarily account for the synergistic effect of the endothelium, pericyte, and astrocyte cells that constitute the barrier, which may miss on important filter mechanisms for EVs. Future in vitro methodologies should consider an assay that includes these cell types in between chambers.

#### EVs origin and labeling for blood–brain barrier studies

Heterogeneity of extracellular vesicles is a major concern when trying to derive generalizable conclusions to experimental results. It has been shown that even different isolation methods used to obtain EVs from the same cell type can result in different proteomic profiles, thus separating two or more different EVs populations [2]. Therefore, comparing results from assays not using the same cells of origin or isolation procedures can be challenging, as it is the case for literature describing BBB crossing by EVs. Table 2 describes the characterization of EVs across the different studies evaluating crossing of the BBB.

**Table 2:** Characterization of extracellular vesicles

Author	Cell of origin	Isolation procedure	Size (nm)	Zeta potential	Positive EV markers	Negative EV markers	Concentration	Total protein
Morad et al	MDA-MB-231	Ultracentrifugation at 100000 g for 90 min at 4 °C	154.1 ± 7.0 and 158.5 ± 6.0	N.A	CD9, CD63, Alix	GM130	1 × 10^11^ particles/mL	N.A
Tominaga et al	MDA-MB-231-luc-D3H1 and MDA-MB-231-luc-D3H2LN	Ultracentrifugation at 110,000 g for 70 min at 4 °C	100	N.A	CD63, CD9	Cytochrome C	1.2 × 10^9^ particles/mL	N.A
Chen et al	HEK 293 T cells	Ultracentrifugation 120,000 g for 2.5 h at 4 °C	96.3 ± 5.4 and 80.3 ± 2.0	N.A	CD63, CD9, CD81	N/A	3 × 10^8^ and 6 × 10^8^ particles/mL	N.A
Matsumoto et al	Human erythrocytes	SEC	205.22 ± 1.79	N.A	Alix	CD235a (RBC)	1.76 × 10^9^ particles/mL	0.68 ± 0.11 mg/dish
Kuroda et al	SK-Mel-28	ExoQuick-TC (polymer based extraction by precipitation) and MagCapture (Affinity method for phosphatidylserine)	217.0 ± 4.5	N.A	CD9,CD81,TSG101,Alix,flotillin-1	calnexin, GRP78	9 × 10^9^ particles/mL	N.A

*EVs* extracellular vesicles, *SEC* Size exclusion column, *N.A.* information not available

Table 3 shows the tissue of origin and labeling techniques for EVs that have been used in the evaluation of BBB crossing. From the described transwell studies, Morad et al. used the human brain-seeking MDA-MB-231 breast cancer cell line that derives from a metastatic pleural effusion site [31]. This group was able to detect EVs crossing to the abluminal side of the monolayer transwell. Tominaga et al. also utilized breast cancer lines MDA-MB-231-luc-D3H1 and MDA-MB-231-luc-D3H2LN, and generated a subset of brain metastatic derivative populations (BMD2a and BMD2b) [29]. However, they were able to detect EVs in endothelial cells but not pericytes of a bilayer transwell, or in astrocytes in the abluminal side of the assay. This highlights the importance of the number of layers used for BBB models, as described previously, even when using similar cells of origin for EVs.

**Table 3:** Tissue origin of extracellular vesicles and techniques used for labeling

Author	Organ of origin	Cell line	Labeling	Result
Morad et al	Breast cancer	MDA-MB-231	Gaussia luciferase; TdTomato	EVs crossed monolayer
Tominaga et al	Breast cancer	MDA-MB-231-luc-D3H1 and MDA-MB-231-luc-D3H2LN	PKH67; PKH26; DiR	Detected in endothelium but not pericytes or astrocytes
Chen et al	Embryonic kidney	HEK 293 T cells	Gaussia luciferase fused with lactadherin; PKH67; PKH26	Crossed only with TNF-α treatment
Matsumoto et al	Parkinson´s disease and healthy control RBC	Human erythrocytes	Na^125^I; Na^131^I; Dil	Crossed only with LPS treatment
Kuroda et al	Melanoma	SK-Mel-28	PKH67	Successful incorporation but not crossing of the endothelium

*RBC* red blood cells, *EVs* extracellular vesicles, *TNF-α* tumor necrosis factor alpha, *LPS* lipopolysaccharide

Chen et al. analyzed EVs from a different cell line, the human HEK 293 T cells of the embryonic kidney epithelium [30]. Even though they employed a monolayer as Morad et al., EVs from these experiments were unable to significantly cross the BBB unless an inflamed environment was simulated with TNF-α treatment (up to approximately 10% of exosomes crossed from the luminal to abluminal chamber after 18 h) [30]. Matsumoto et al. isolated EVs from human red blood cells (RBC) instead, both from Parkinson's disease and healthy control patients [28]. Consistent with Chen et al., they showed that despite being largely impermeable to RBC-EVs under healthy conditions, the BBB monolayer model allowed increased crossing of EVs after administration of lipopolysaccharide (LPS) to mimic inflammation [28]. Finally, although not a transwell, Kuroda et al. evaluated successful incorporation, but not crossing, of the melanoma cell line SK-Mel-28 PKH67-labeled EVs into human blood − brain barrier endothelial hCMEC/D3 cells after incubation at 37 °C [32].

Techniques used so far to label and visualize EVs through BBB models vary (Table 3). Morad et al. used *Gaussia* luciferase for the transwell models and palmitoylated TdTomato for incubation with BMECs, after cancer cell transduction with lentiviral vectors [31]. *Chen *et al*.* labeled EVs with humanized *Gaussia* luciferase (hGluc) fused with lactadherin, also through a lentiviral vector on 293 T cells [30]. Marking of EVs to evaluate uptake into BMECs was done utilizing PKH67 and PKH26 dyes as well [30]. These last two were also used by Tominaga et al*.* for labeling cancer cells EVs in BBB in vitro assays [29]. Nevertheless, in vivo experiments were conducted by tagging EVs with DiR, a lipophilic near-infrared fluorescent cyanine dye [29].

Kuroda et al. employed PKH67 for assessing EVs internalization [32]. Alternatively, the method of choice by Matsumoto et al. was radioactive labeling of RBC-EVs with Na^125^I or Na^131^I by chloramine T [28]. Lastly, for immunofluorescence staining after intravenous injection, RBC-EVs were labeled with DiI cell-labeling solution [28].

Results from transwell experiments differ considerably depending on the EV cell of origin and the cell layers in the assay construction. Those originating from breast cancer showed internalization in endothelial cells, pericytes, and astrocytes independently, but could not cross beyond the endothelium when faced with more than one layer [29]. Studies using EVs derived from the HEK 293 T cell line and human erythrocytes demonstrated crossing of a BBB monolayer only when associated with inflammation [28, 30].

The increase in EVs permeability under inflammatory conditions appear to favor an active transcellular transport rather than passive paracellular diffusion [28, 30]. Matsumoto et al. showed an LPS dose-dependent decrease in TEER, that reached a reduction of approximately 75% at 100 ng/mL [28]. The permeability of EVs increased almost 300% relative to vehicle controls after treatment with an LPS dose of 100 ng/mL [28]. Pharmacokinetic studies in mice showed that the unidirectional influx constant (Ki) of RBC-EVs was not significantly different compared to albumin in normal conditions [28]. However, under LPS treatment, the Ki of mice was significantly higher for RBC-EVs compared to the much smaller albumin molecule, which suggests that RBC-EVs cross the BBB more easily (LPS; 0.5533 ± 0.1704 μL/g-min vs control; 0.1079 ± 0.05487 μL/g-min, p = 0.0199) [28].

Results with TNF-α treatment from Chen et al. support the idea of an active transcellular transport mechanism of EVs in inflammatory conditions [30]. Most interestingly, no significant differences in bioluminescence activity were observed for paraformaldehyde-fixed BMECs under both TNF-α activated and untreated conditions at all time points, unlike the much greater exosome crossing of the living BMECs under TNF-α (up to approximately 10% of EVs) compared to untreated condition [30]. Immunofluorescence of VE-cadherin, ZO-1, and Claudin-5 showed that their expression levels were significantly down-regulated after TNF-α treatment, which also plays a role in the altered intercellular permeability of BMECs [30].

Labeling techniques include several approaches to be used in different contexts. Fluorescent proteins expressed in the EV producer cell, such as Tdtomato having a red emission, allows an optimal visualization for in vitro or ex vivo experiments. However, due to the heterogeneity in the EV population obtained through the different separation methodology used, the standardization of the loaded fluorescent protein inside the EVs must be contemplated for a correct application. Similarly, lipophilic fluorophores such as PKH67, PKH26, DiI, and DiR give an appropriate tagging to be observed at in vitro or ex vivo settings. On the other hand, radioactive isotopes enable better in vivo visualization due to their higher tissue penetration, allowing in turn the ex vivo tracking by histological analyzes or in vitro experiments. Nevertheless, both the lipophilic fluorophores and the radionuclide labeling are incorporated into the EV lipid bilayer [28–32, 36]. Hence, it must be considered that those labeling strategies imply the EV structure modification after its production and isolation. Therefore, to avoid artifacts in the obtained images, it is imperative to use a suitable methodology to separate the free fluorophore, radionuclide, or released dye from the tagged EVs. Moreover, physicochemical analysis and functionality evaluations of tagged EVs must be performed to assure the invariability of EV characteristics. The use of Gaussia luciferase or other luciferase enzyme system permits an in vivo evaluation of the EV distribution due to the NIR emission without using an external excitation source. This also allows the ex vivo and in vitro trafficking evaluations. Nevertheless, the principal limitation to understanding the biodistribution and the mechanisms that enable EVs movement is that none of the mentioned reporter systems can guarantee the integral EV structure when it passes through biological barriers. Therefore, it is an important challenge to determine the fate of the cargo and EVs after the uptake and during the proposed transcytosis.

### Evaluation of uptake and transcytosis

**Fig. 1:**
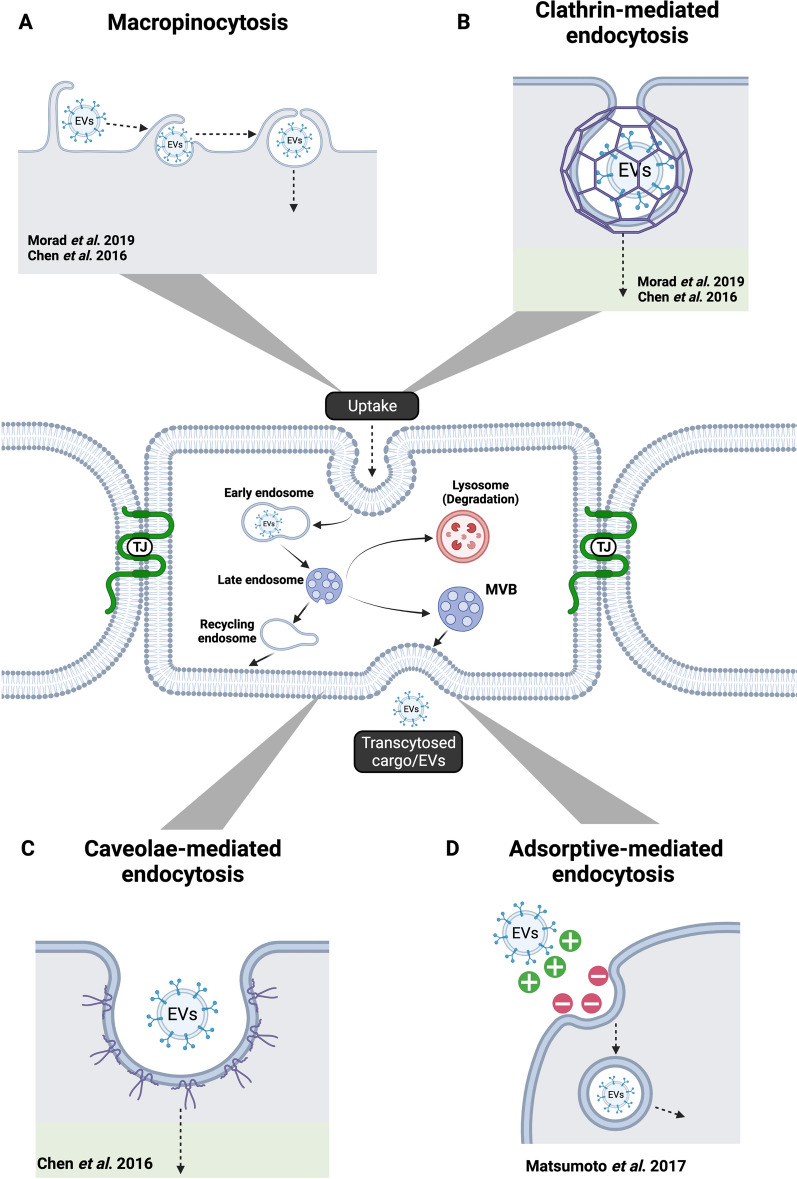
Uptake and transcytosis mechanisms for crossing of extracellular vesicles through the BBB. The figure shows four uptake mechanisms that have been evaluated and proposed for the active transport of EVs across the BBB and the authors that have described evidence to support them: **A** Macropinocytosis [30, 31]. **B** Clathrin-mediated endocytosis [30, 31]. **C** Caveolae-mediated endocytosis [30]. **D** Adsorptive-mediated endocytosis [28]. The fate of EVs after internalization include recycling to the plasma membrane, degradation of EVs by lysosomes, and final transcytosis of EVs and their cargos to the extracellular space. *EVs* extracellular vesicles, *BBB* blood brain barrier, *MVB* multivesicular body, *TJ* tight junctions

Uptake refers to the internalization pathways of particles through the cell membrane that usually occur through pinocytosis, which can be subcategorized into clathrin-mediated endocytosis, caveolae-mediated endocytosis, clathrin- and caveolae-independent endocytosis and micropinocytosis (Fig. 1) [37]. Following uptake, intracellular trafficking of particles, including EVs, will determine their destination within cellular compartments [37]. Some will encounter degradation after integrating with lysosomes [37]. However, some will escape this pathway with the possibility of cellular release (Fig. 1) [37].

Transcytosis is the transport of macromolecular cargo from one side of a cell to the other within a membrane-bounded carrier(s) (Fig. 1) [38]. Transport pathways of substances across the BBB include paracellular diffusion, transcellular diffusion, protein-mediated transport (such as GLUT-1, CNT2, and MCT1), receptor-mediated transcytosis, and adsorptive-mediated transcytosis [39, 40]. From these mechanisms, active transcytosis has been suggested as the most likely in EVs crossing of the BBB [39]. Studies by Chen et al. and Morad et al. have shed light on specific processes that might be involved. Both identified a significant reduction of EVs internalization or crossing through brain endothelial cells at 4 °C, indicating that an active mechanism should be responsible for transport. Morad et al. further treated with Dynasore, an inhibitor of endocytosis, decreasing the rate of EVs detected in abluminal chambers of transwell analyses [31].

Using uptake inhibition techniques associated with measurements by flow cytometry from cultured BMECs in 12-well plates exposed to labeled EVs, as well as assays evaluating colocalization of EVs to specific proteins, several endocytic pathways have been examined (Table 4; Fig. 1): macropinocytosis, clathrin-dependent endocytosis, and lipid raft/caveolae-dependent endocytosis [30, 31].

**Table 4:** Uptake inhibition techniques used to evaluate endocytic pathways

Mechanism	Author	Cells and EVs	Inhibitor (concentration)	Pre-treatment duration	Incubation with EVs	Uptake of Evs
Macropinocytosis	Morad et al	BMECs + TdTom-Br-EVs	EIPA (100 μM); cytochalasin D (500 nM)	30 min	3 h	Decreased
	Chen et al	BMECs + PKH26-labeled exosomes	EIPA (1 mM); cytochalasin D (20 μM)	30 min	1 h	Decreased
Clathrin-dependent endocytosis	Morad et al	BMECs + TdTom-Br-EVs	Chlorpromazine (20 μM); ML141 (100 μM)	30 min	3 h	Decreased
	Chen et al	BMECs + PKH26-labeled exosomes	Chlorpromazine (15 μM)	30 min	1 h	Decreased
Lipid raft/caveolae-dependent endocytosis	Morad et al	BMECs + TdTom-Br-EVs	Filipin III (10 μM)	30 min	3 h	No effect
	Chen et al	BMECs + PKH26-labeled exosomes	Filipin III (5 μM); MβCD (5 mM); nystatin (5 μM)	30 min	1 h	Decreased

*EIPA* 5-(*N*-ethyl- *N*-isopropyl) amiloride, *MβCD* methyl- β-cyclodextrin

Morad et al*.* used 5-(*N*-ethyl- *N*-isopropyl) amiloride (EIPA) and cytochalasin D to block macropinocytosis, which decreased the uptake of EVs significantly (approximately a 50% and 75% reduction relative to control, respectively) [31]. This was supported by TdTom-EVs partially colocalizing with 70 kDa dextran, a marker for macropinocytosis, under fluorescence microscopy images [31]. Similarly, Chen et al. treated with the same inhibitors and their results concurred under TNF-α inflammatory conditions after 18 h incubation (approximately an 80% and 45% reduction relative to control, for EIPA and cytochalasin D respectively). Inhibition was assessed by EVs uptake assay using confocal microscopy and image analysis [30].

Clathrin-dependent endocytosis was inhibited by chlorpromazine and a Cdc42/Rac1 GTPase inhibitor, ML141, in Morad et al. studies [31]. These were able to decrease EVs uptake significantly (approximately a 40% and 60% reduction relative to control, for chlorpromazine and ML141 respectively). TdTom-EVs also colocalized with Alexa647 transferrin, a marker of this pathway. In agreement with these findings, Chen et al*.* evidenced an attenuation of EVs uptake when blocking with chlorpromazine alone (approximately a 55% reduction in the native condition and a 71% reduction in the TNF-α condition, relative to control) [30].

However, the two groups differ on their results for lipid raft/caveolae-dependent endocytosis experiments. Morad et al. could not identify a role of this route, as inhibition by filipin showed no effect on EVs uptake in endothelial cells by flow cytometry [31]. There was also a lack of colocalization of EVs with caveolin. In contrast, Chen et al*.* pre-incubated BMECs with cholera toxin B (CtxB), a late endosomal compartment marker, and found a decrease of PKH67-labeled exosomes uptake by BMECs after treatment with filipin III (approximately a 27% reduction in the native condition and a 64% in the TNF-α condition, relative to control) [30]. They observed the same results with two additional inhibitors: methyl- β -cyclodextrin (approximately a 51% reduction in the native condition and a 61% reduction in the TNF-α condition, relative to control) and nystatin (approximately a 37% reduction in the native condition and a 46% reduction in the TNF-α condition, relative to control) [30]. Therefore, the authors concluded that caveolae-dependent endocytosis is one likely route of EV internalization [30].

Other positive colocalization results by Morad et al*.* included Rab11 (a marker of recycling endosomes), BODIPY conjugated DQ-ovalbumin (a marker of endolysosomal structures), VAMP-3 (marker of exocytosis and recycling), VAMP-7 (marker of lysosome fusion), and SNAP23/Syntaxin 4 (complex on the basolateral membrane) [31]. These indicate that EVs fate after internalization can include recycling, transcytosis, or degradation (Fig. 1). Chen et al*.* did not evaluate these parameters.

Antibody and knockdown strategies have also been used to test for the influence of surface receptors of BBB endothelial cells on exosome uptake. Kuroda et al. utilized anti-integrin α5 and anti-integrin αV antibodies, which were able to reduce the uptake by 11.8% [32]. Interestingly, CD46 small interfering RNA (siRNA) transfection into hCMEC/D3 endothelial cells also revealed a 39.0% decrease of exosome uptake [32].

Transcytosis might be the primary active mechanism for EVs crossing the healthy BBB [39]. There is evidence that macropinocytosis and clathrin-dependent endocytosis have a role even in EVs originating from different cell types [30, 31]. However, lipid raft/caveolae-dependent endocytosis shows inconsistent data depending on the technique used and EVs' originating cell line. As only two groups have examined this specific problem, differences in the data could also be model dependent.

Given the intracellular processes that involve uptake and transcytosis, without live-imaging and proper tracing, it is difficult to determine whether the same EVs from the discussed assays permeate across the BBB. It cannot be excluded that the contents from the internalized EVs are released from the original lipid bilayer, repacked in structures such as multivesicular bodies (MVB), and again released as EVs. In addition, the mere identification of a dye or fluorescent marker on the opposite side of a transwell assay does not guarantee its association to the lipid bilayer of an EV.

### In vivo models for EVs crossing of the blood–brain barrier

#### Human and mouse models

The following section will describe evidence in mouse models and human research participants that indicates EVs can somehow move in a bidirectional manner through the BBB. However, there is only indirect data to suggest the mechanisms of transcytosis. Alzheimer's studies in humans, for instance, have revealed that exosomes with Central Nervous System (CNS) components can be detected in the peripheral blood and have emerged as potential biomarkers [41–45]. Morad et al*.* performed retro-orbital injections of TdTom-Br-EVs isolated from brain-seeking MDA-MB-231 breast cancer cells and evaluated the distribution of EVs to the brain in nude mice [31]. Histological analyses showed that Br-EVs were taken up by glial fibrillary acidic protein (GFAP) + astrocytes. Shi et al. were able to detect exosomes carrying CNS α-syn in blood, which is correlated with Parkinson’s disease, by an immunoaffinity capturing protocol that isolates L1 cell adhesion molecule (L1CAM)-containing exosomes in human or mouse plasma [46]. However, it is worth mentioning that a recent report by Norman et al. has recommended against the use of L1CAM as a marker in neuron-derived EVs isolation protocols as they demonstrated no association between L1CAM with EVs in human cerebrospinal fluid or plasma [47]. Nonetheless, Shi et al*.* discuss that exosomes possibly cross multiple layers of the BBB by jumping from cell-to-cell via the MVB compartment, citing a theory by Record et al. that relates exosomes to processes observed in HIV, but do not test the hypothesis experimentally in their model [46, 48–50].

Adsorptive mediated transcytosis (AMT) was proposed by Matsumoto et al. after analyzing the co-injection of unlabeled RBC-EVs and labeled ^125^I-RBC-EVs in CD1-mice (Fig. 1) [28]. As increasing doses of unlabeled EVs did not affect LPS-induced entry of labeled EVs, they concluded that probably the transfer mechanism does not occur via a saturable process [28]. The authors further examined the influence of wheat germ agglutinin (WGA), a potent AMT inducer, which increased brain uptake of ^125^I-RBC-EVs relative to controls [28]. Nevertheless, RBC-EVs were found to colocalize with antibodies against Iba-1-labeled microglia, but not GFAP-labeled astrocytes or MAP2-labeled neurons [28].

Another method that has been utilized to evaluate the EVs transport from the brain to peripheral blood, and therefore indirectly proving crossing of the BBB, is orthotopic xenotransplantation in mice of human tumor cells [51]. García-Romero et al*.* were able to isolate human gDNA from EVs originated from glioblastoma-cancer stem cells (GBM27 and GBM38), circulating in the bloodstream after transplantation. However, they did not address possible mechanisms of transcytosis at the level of the BBB [51]. The mouse model of Tominaga et al. did not analyze transcytosis in vivo either but showed promotion of cancer cell metastasis by EVs through the destruction of the BBB by miR-181c and its target gene downregulation, PDPK1 [29]. This could also play a role in the crossing of EVs populations in cancer.

Banks et al*.* surveyed EVs from 10 different sources, including six cancer and four non-cancerous cell lines in CD-1 mice [52]. These included EVs from mice macrophages (J774A.1), fibroblasts (NIH-3T3), and oral squamous cells (SCCVII), as well as human T cells (primary T cell), keratinocytes (HaCaT), melanoma (MEL526), breast (MDA-MB-231), head and neck cancer cells (PCI-30 and SCC-90), and leukemia (Kasumi) [52]. They used the capillary depletion and the intracerebroventricular injection methods to determine, through radioactive 0.5 mCi ^125^I labeling of EVs, the degree to which EVs crossed the BBB from the peripheral blood compartment or the brain-to-blood efflux rate, respectively [52].

All EVs tested in Banks et al. analyses were reported to cross the BBB with different influx rates [52]. Neither species nor cancer state seemed to influence the uptake [52]. For additional characterization, LPS, WGA, and mannose 6-phosphate (M6P) impact on uptake were measured [52]. LPS increased uptake of six human EVs populations and decreased uptake from one murine type [52]. AMT uptake appeared to increase in half of the EV types (J774A.1, NIH-3T3, HaCAT, SCC-90, Kasumi) exposed to WGA [52]. In contrast, M6P blocked uptake of fibroblast EVs, exhibiting dependence on the mannose 6-phosphate receptor for transport of this type of EVs [52]. Despite this relevant description of EVs crossing compartments, no direct visualization of transcytosis through the BBB was executed.

#### Zebrafish model

To date, there is only one study that evaluates in vivo crossing of EVs through a direct study of the BBB. Morad et al. developed a Tg(kdrl:GFP) zebrafish model from embryos incubated in E3 medium at 28.5 °C [31]. Experiments were performed 6–7 days postfertilization, when an intracardiac injection of TdTom-Br-EVs (5 nL of a 400 μg/mL suspension per injection) was done using the Narishige Injection System [31]. One hour post-injection, live imaging of embryos was conducted using a Nikon Eclipse Ti inverted microscope with a Yokogawa spinning disk scan head and an Andor iXon EM-CCD camera [31]. Integrity of the BBB was verified by intracardiac injections of unlabeled Br-EVs (60 μg/mL), 10 kDa dextran-Alexa Fluor 647, and 70 kDa rhodamine B-dextran (60 μg/mL) [31].

This group was able to detect Br-EVs in the brain parenchyma at the time of imaging and time-lapse showed movement of EV-containing endocytic vesicles within endothelial cells [31]. Some vesicles were able to reach the plasma membrane and fuse with it. The integrity of the BBB was preserved against 10 kDa and 70 kDa dextran [31]. Together, this evidence demonstrated crossing of EVs through the BBB in a process suggestive of transcytosis.

**Fig. 2:**
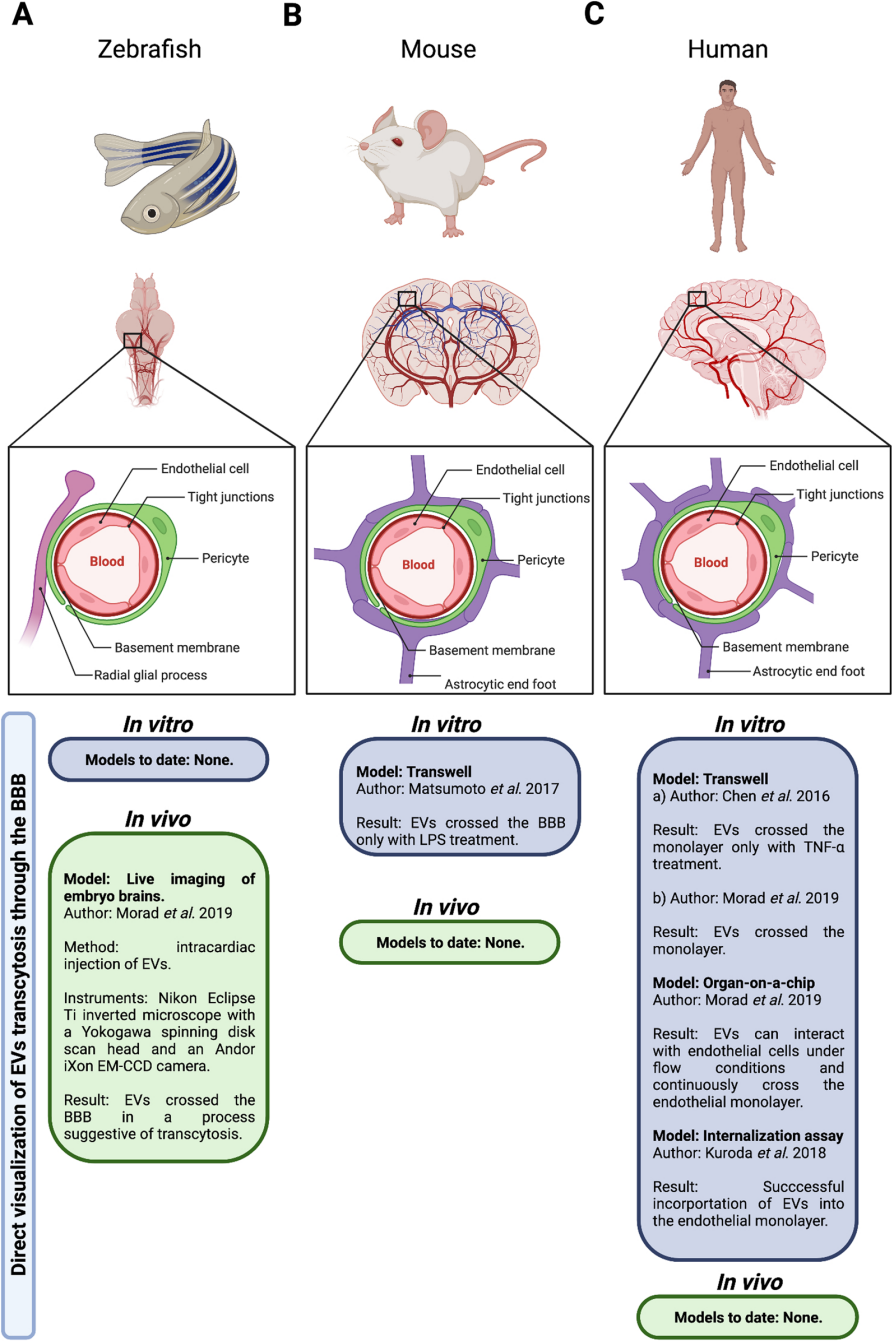
Comparison of BBB structures of zebrafish, mice, and humans. The figure shows the main components of the BBB, including endothelial cells with specialized tight junctions, pericytes, and astrocytic or glial processes. **A** The zebrafish BBB has a less complex neurovascular unit that lacks classic stellate astrocytes, with radial glial processes that rarely become in contact with the vasculature. **B** The mouse BBB presents astrocytic end feet in close contact with the vasculature. **C** Compared to mice, the human BBB shows a greater number of astrocytic end feet. Also shown are the in vitro and in vivo models that have evaluated direct visualization of EVs transcytosis through the BBB in these species. *EVs* extracellular vesicles, *BBB* blood brain barrier, *EM-CCD* electron multiplication charge-coupled device, *TNF-α* tumor necrosis factor alpha, *LPS* lipopolysaccharide

The zebrafish has been described as a suitable model for BBB studies [53, 54]. However, there are several factors that need to be considered before drawing conclusions on mammals based on this model (Fig. 2). Endothelial cells at the BBB are indeed comparable as demonstrated by conserved genetic expression of tight junction molecules such as ZO-1 and Cldn5, as well as the glucose transporter Glut1, the efflux pump Pgp, and the transcytosis inhibitor Mfsd2a [36, 53, 55, 56]. Nevertheless, zebrafish pericytes do not express canonical mammalian markers such as Rgs5a, Desmin a/b, or Cspg4, and originate from both neural crest and mesenchyme, rather than just from neural crest as in mice and humans [57–66].

Another critical difference between mammalian and zebrafish BBB is the astrocyte component (Fig. 2). Zebrafish do not possess classic stellate astrocytes [57]. Instead, they express radial glia lacking polarization of Aqp4, with processes that rarely become in contact with the vasculature [53, 57, 67]. This could contribute to an impaired BBB function compared to mammals. Zebrafish is considered a more ancestral endothelial BBB with a less complex neurovascular unit [57]. Therefore, more studies are still needed to determine if EVs can surpass the BBB in mammals in vivo as they do in zebrafish.

### Future perspectives for EVs evaluation through the BBB

New techniques have been developed to analyze the physiological characteristics and dynamics of tissues that, if applied to this problem, could offer a better understanding of the crossing of EVs through the BBB. These include BBB organoids and microfluidic platforms.

As previously described, Transwell assays face many challenges, including irregularities in cellular cultures that can produce leakiness or multiple cell layers [68, 69]. Endocytic events from the basolateral site can be confused with exocytosis, and transcytosis might be mistaken by crossing of EVs through a hole nearby the cell [69]. These problems can be overcome by applying live-cell imaging techniques such as spinning disc confocal microscopy or total internal reflection fluorescence microscopy (TIRFM) to exclude confounding transport through imperfections [70].

Organoids of the BBB could offer a more complete and realistic model to evaluate the barrier crossing potential of EVs. Spheroid models of the BBB have already been proposed for investigating CNS therapeutics, but not in the context of EVs [68, 71, 72]. These have used endothelial cells, pericytes, and astrocytes with detection strategies based on confocal microscopy for fluorescently labeled compounds and MALDI mass spectrometry imaging for nonfluorescent molecules [68]. Future in vitro analyses with EVs should consider more advanced structures of the BBB in the form of organoids assembled with a scaffolding extracellular environment and adding layers of endothelium, pericytes, astrocytes, and neurons to the model. Several teams have already developed 3D models of microfluidic spheroid triple-cultures consisting of human brain endothelial cells, pericytes, and astrocytes that could meet these requirements for EVs analysis [71, 73, 74].

On their own, however, organoids cannot mimic fluid flow and shear stress. Therefore, they must be complemented with microfluidic platforms or dynamic in vitro models. 3D printing technology has revolutionized the field allowing the construction of complex structures with materials such as collagen and hydrogels [75]. These microenvironments have been recreated by Brown et al., Tourovskaia et al., and Vernetti et al., including microvascular endothelial cells, astrocytes, and pericytes for BBB simulation [76–78]. Nevertheless, differences in protein expression in the artificial matrix compared to the in vivo extracellular matrix, reduction of homogeneity and reproducibility, and lack of complete replication of metabolic mechanisms are still limitations of these systems [75].

In vivo experiments must be added to the selection of methodologies described to accomplish the best characterization of the crossing or transcytosis events of EVs through the BBB components towards neurons. In this regard, classical EV-labeling techniques such as bioluminescence (luciferase), fluorescent proteins (e.g. GFP and RFP), and organic fluorescent dyes (e.g. DiR, DiD, PKH67, PKH26, R18, Alexa Fluor, CFDA-SE, and calcein AM) could be coupled with intravital techniques such as fast high-resolution miniature two-photon microscopy (FHIRM-TPM) for brain imaging [79–81]. This could offer real-time visualization of EVs distribution from the periphery, through the BBB, and to the brain parenchyma in living mammals.

In the same way, complementary biodistribution imaging of EVs through the system may be assessed using magnetic resonance imaging (MRI), single-photon emission computed tomography (SPECT), or positron emission tomography (PET) [79]. Superparamagnetic iron oxide nanoparticles have been used in tracking EVs with the application of MRI due to their reduction of T2 signal as a contrast relative to the surrounding tissues [79]. Furthermore, to avoid misinterpreting tracer signals on in vitro*, *ex vivo*,* or in vivo EV tracking experiments, it is imperative to use validated methodologies to separate the free dye from labeled EVs appropriately. In addition, due to label leakage, membrane destabilization, and/or EV fusion with the recipient cell bilayer or organelles, it should be desirable to use a double labeling approach. This will allow the colocalization of two signals, one in the lipophilic space, such as with an organic fluorescent dye, and another bioluminescence enzyme system or a nanoparticle marker in the hydrophilic space, assuring the EV structure integrity once the visualization experiment is performed. Another possibility for characterization in this context could be Correlative Light and Electron Microscopy (CLEM) or Cryo-CLEM [82, 83]. This will allow a dynamic description of the motion of EVs from the periphery, through the BBB, and to specific anatomical structures of the CNS, although the precise mechanisms of transcytosis would have to be evaluated as previously described.

#### Alternative pathways

**Fig. 3:**
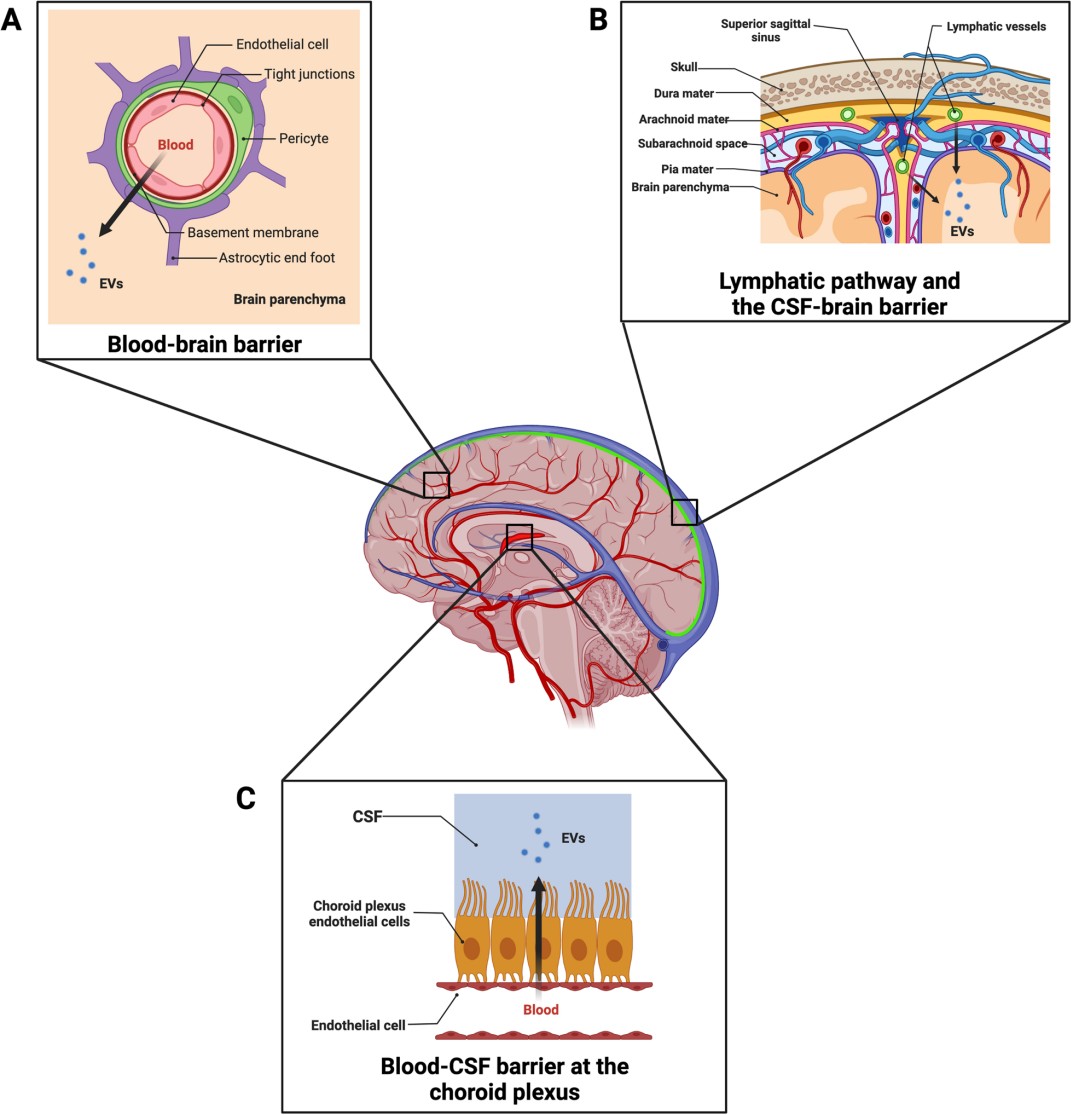
Anatomical pathways and barriers of the CNS as potential routes for extracellular vesicles. The figure shows theoretical vascular points of entrance for EVs and checkpoint barriers to the brain parenchyma that should be further examined. **A** The classical path that has been evaluated for EVs crossing from peripherical blood to the brain is through arterial flow and the BBB, comprised of endothelial cells, pericytes, and astrocytes [28, 30–32]. **B** The recently discovered meningeal lymphatic vessels expose a route to the CSF-brain barrier not yet explored and that EVs could exploit to access the brain parenchyma [85]. **C** A third entrance point is through the Blood-CSF barrier at the choroid plexus [84]. EVs: extracellular vesicles; BBB: blood brain barrier; CNS: Central Nervous System; CSF: cerebrospinal fluid

Evidence has emerged on the role of exosomes in the transport of micronutrients, such as 5-methyltetrahydrofolate (5MTHF), through the blood-cerebrospinal fluid barrier at the choroid plexus (Fig. 3) [84]. Grapp et al. injected exosomes derived from folate receptor α (FRα)-transfected Z310 cells (an immortalized rat choroid plexus cell line) into the lateral ventricle of C57BL/6 mice [84]. By labeling exosomes using the PKH26 dye and through immunohistochemistry analysis, they were able to show that exosomes penetrated the brain parenchyma and colocalized with GFAP-positive astrocytes as well as NeuN-positive neurons [84]. They suggest a clathrin-independent pathway through glycosylphosphatidylinositol-anchored protein-enriched endosomal compartments that promote FRα translocation to multivesicular bodies before being released as exosomes [84]. This pathway at the choroid plexus should be further examined as an EVs transportation site to the CNS.

A final note that is currently being evaluated by our team is the possible bidirectional motion of EVs from brain and peripheral tissues through the recently discovered meningeal lymphatic vessels (Fig. 3). Brain lymphatics were discovered in the meningeal spaces parallel to the dural sinuses expressing all the molecular hallmarks of lymphatic endothelial cells [85]. The system allows for the carrying of both fluid and immune cells from the cerebrospinal fluid and is connected to the deep cervical lymph nodes [85]. Given their initial type characteristics, the meningeal lymphatic pathway might represent a less stringent barrier for EVs crossing compared to the classical BBB associated with blood vessels. This should be further evaluated as an additional mechanism for EVs crossing to and from the CNS.

## Conclusions

Evs crossing of the BBB in a bidirectional manner between the bloodstream and brain parenchyma remains poorly understood. Most in vitro models that have evaluated this event have relied on monolayer transwell or microfluidic organ-on-a-chip techniques that do not account for the combined effect of all cellular layers that constitute the BBB at different sites of the CNS. Some authors have described EVs internalization by all BBB cell types but not crossing beyond the endothelium when EVs face a multilayer challenge [29]. Others have suggested that an inflammatory environment is needed for crossing [28, 30]. Notably, research is limited to analysis of BBB crossing by EVs originating from peripheral or cancer tissue. However, studies of EVs deriving from pericytes, astrocytes, or neurons are missing in the evaluation of uptake and transcytosis through the BBB.

Transcytosis might be the primary active mechanism for EVs passing through healthy BBB. There is evidence that macropinocytosis and clathrin-dependent endocytosis have a role even in EVs originating from different cell types [30, 31]. However, lipid raft/caveolae-dependent endocytosis shows conflicting data depending on the technique used and/or EV originating cell line [30, 31].

There has not been direct visualization of transcytosis through the BBB in mammals, including humans. Evidence of transcytosis comes from in vivo experiments in zebrafish. However, pericytes, astrocytes, and endothelial cells in zebrafish lack characteristics of the more complex neurovascular unit in mammals. Therefore, future in vitro research should consider the use of organoids to model transcytosis of the BBB, complemented with microfluidic and dynamic platforms to mimic flow and shear stress. In vivo studies should include EVs labeling coupled with intravital techniques such as FHIRM. Complementary biodistribution imaging of EVs through the system may be assessed using MRI, SPECT, or PET.

## Data Availability

Not applicable.

## Abbreviations

AMT: Adsorptive mediated transcytosis
BBB: Blood–brain barrier
BMEC: Brain microvascular endothelial cell
CNS: Central Nervous System
CtxB: Cholera toxin B
EIPA: 5-(*N*-Ethyl- *N*-isopropyl) amiloride
EM-CCD: Electron multiplication charge-coupled device
EVs: Extracellular vesicles
FHIRM-TPM: Fast high-resolution miniature two-photon microscopy
FITC: Fluorescein isothiocyanate
GM-CSF: Granulocyte–macrophage colony-stimulating factor
hGluc: Humanized *Gaussia* luciferase
L1CAM: L1 cell adhesion molecule
LAMP1: Lysosome-associated membrane protein 1
LPS: Lipopolysaccharide
MRI: Magnetic resonance imaging
M6P: Mannose 6-phosphate
MAGE-3: Melanoma-associated antigen 3
MβCD: Methyl- β-cyclodextrin
MVB: Multivesicular body
PET: Positron emission tomography
RBC: Red blood cells
SPECT: Single photon emission computed tomography
siRNA: Small interfering RNA
TIRFM: Total internal reflection fluorescence microscopy
TEER: Transendothelial electrical resistance
WGA: Wheat germ agglutinin
